# Trends in Industry Payments to Physicians in the First 6 Years After Graduate Medical Training

**DOI:** 10.1001/jamanetworkopen.2022.37574

**Published:** 2022-10-19

**Authors:** Misop Han, Sean O. Hogan, Eric Holmboe, Yuezhou Jing, Kenji Yamazaki, Bruce J. Trock

**Affiliations:** 1 James Buchanan Brady Urological Institute, Department of Urology, Johns Hopkins School of Medicine, Baltimore, Maryland; 2 Accreditation Council for Graduate Medical Education, Chicago, Illinois

## Abstract

**Importance:**

Financial incentives and conflicts of interest may influence physician decision-making. It is important to understand financial interactions between the pharmaceutical and medical device industries and newly independent physicians who have recently completed their graduate medical education using a national transparency program.

**Objective:**

To identify trends in industry payments to recent graduates of Accreditation Council for Graduate Medical Education–accredited residency or fellowship programs in orthopedic surgery, neurosurgery, and internal medicine.

**Design, Setting, and Participants:**

This retrospective cohort study analyzed Open Payments reports of industry payments made between July 1, 2015, and June 30, 2021, to newly independent physicians from residency or fellowship programs in neurosurgery, orthopedic surgery, and internal medicine who graduated between January 1, 2015, and December 31, 2019.

**Exposures:**

Specialties (neurosurgery and orthopedic surgery, with internal medicine as a comparison group).

**Main Outcomes and Measures:**

Industry payments to newly independent physicians, including any general payments (noninvestment or nonresearch) and at least $5000 of general payments in aggregate value per year, which are considered significant financial conflicts of interest. The percentage of newly independent physicians accepting general payments during the first 6 years after graduation was analyzed by specialty and sex using cumulative incidence curves and hazard ratios (HRs) in univariable and multivariable analyses.

**Results:**

There were 45 745 recent graduates (28 137 men [62%]; median age at graduation, 33.0 [IQR, 31.0-35.0 years]) in neurosurgery (n = 595), orthopedic surgery (n = 3481), and internal medicine (n = 41 669). In the first 2 years of independent practice, 95% (n = 3297), 92% (n = 546), and 59% (n = 24 522) of newly independent physicians in orthopedic surgery, neurosurgery, and internal medicine, respectively, accepted any general payments. A higher percentage of the newly independent physicians in orthopedic surgery and neurosurgery accepted any general payments (orthopedic surgery vs internal medicine: HR, 5.36 [95% CI, 4.42-6.51] for women and 7.01 [95% CI, 6.35-7.73] for men; neurosurgery vs internal medicine: HR, 3.25 [95% CI, 2.24-4.72] for women and 4.08 [95% CI, 3.37-4.94] for men; *P* = .03). A higher percentage of male physicians compared with female physicians accepted any general payments (orthopedic surgery, 2884 of 3026 [95%] vs 413 of 455 [91%]; *P* < .001; neurosurgery, 466 of 502 [93%] vs 80 of 93 [86%]; *P* = .01; and internal medicine, 15 462 of 24 609 [63%] vs 9043 of 17 034 [53%]; *P* < .001) and at least $5000 of general payments (orthopedic surgery, 763 of 3026 [25%] vs 71 of 455 [16%]; *P* < .001; neurosurgery, 87 of 502 [17%] vs 5 of 93 [5%%]; *P* < .001; and internal medicine, 882 of 24 609 [4%] vs 210 of 17 034 [1%]; *P* < .001).

**Conclusions and Relevance:**

In this cohort study of newly independent physicians in orthopedic surgery, neurosurgery, and internal medicine, the financial relationship with potential conflicts of interest between newly independent physicians and industry began to develop soon after training programs and continued to expand in the early years of newly independent physician practice. Newly independent physicians in surgical specialties and male physicians accepted significantly higher industry payments. Further studies are needed to evaluate whether modifiable factors are associated with the future outcome of newly independent physicians accepting general payments.

## Key Points

**Question:**

How long after completing their graduate medical training do physicians develop financial relationships with the pharmaceutical and medical device industries?

**Findings:**

In this cohort study including 45 745 physicians in orthopedic surgery, neurosurgery, and internal medicine (as a comparison group), financial relationships and significant financial conflicts of interest formed and expanded in the early years of independent physician practice. Surgical specialty physicians and male physicians accepted higher industry payments.

**Meaning:**

This study suggests that the financial relationship between newly independent physicians and industry developed in the early years of independent physician practice, with surgical specialty physicians and male physicians accepting higher industry payments.

## Teaser

This cohort study examines trends in pharmaceutical and medical device industry payments to recent graduates of Accreditation Council for Graduate Medical Education–accredited residency or fellowship programs in orthopedic surgery, neurosurgery, and internal medicine.

## Introduction

Financial incentives and conflicts of interest have been considered important elements that may influence physician decision-making and may contribute to an increase in health care costs.^1,2,3^ Although physicians and researchers collaborate with the pharmaceutical and medical device industry to develop products to benefit patients, increasing concerns have been raised that these relationships may affect the cost of drugs and medical devices, the quality of patient care, the integrity of scientific investigations, medical education, and clinical practice recommendations.^4,5,6,7,8,9,10,11^ Substantial efforts have been made to identify, limit, and manage potential conflicts of interest in financial relationships between physicians and industry to protect the integrity of professional judgment and to preserve public trust.^4^

Originally implemented under section 6002 of the Patient Protection and Affordable Care Act in 2013, the Open Payments program (OPP), also known as the *Physician Payments Sunshine Act*, is a federally mandated national disclosure program that promotes a more transparent and accountable health care system.^12^ The OPP houses a publicly accessible database of payments to physicians and teaching hospitals that reporting entities, such as pharmaceutical and medical device manufacturers, submit annually to the Centers for Medicare & Medicaid Services (CMS).^12^ The payments in the OPP are reported across 3 major categories: (1) general payments: payments or other transfers of value made that are not in connection with a research agreement or research protocol (eg, travel or lodging, consulting or speaking fees, or entertainment); (2) research payments: payments or other transfers of value made in connection with a formal research agreement or research protocol; and (3) physician ownership or investment interest: information about the ownership or investment interests that physicians or their immediate family members have in the reporting entities.^13^ The annual total dollar value of payments in the OPP, including general payments, research payments, and ownership or investment interest, increased steadily from $8.61 billion in 2014 to $10.03 billion in 2019.^12^ Between 2014 and 2020, the total value of the general payments paid to physicians was $14.78 billion. With the enforcement of and significant civil monetary penalties levied against violations or failure to accurately report payments, OPP reporting is expected to be robust.^14,15^

Studies of the OPP database revealed that a small subset of physicians accepted from industry an unduly significant proportion of general payments, which are nonresearch and noninvestment payments.^16,17,18^ For example, surgical specialty physicians, such as those in orthopedic surgery and neurosurgery, and male physicians accepted higher payments.^17,19,20^ However, most studies on the OPP so far have examined the prevalence of financial relationships (both preexisting and new relationships) between industry and all active physicians, combining data from both newly independent physicians and more established physicians. However, it was unknown how long it takes for the financial relationship to develop between industry and newly independent physicians who recently graduated from their residency or fellowship. Also unknown was the extent of the financial relationship between industry and newly independent physicians. Finally, the role of physicians’ age in accepting industry payments has never been studied, to our knowledge. In this study, we evaluated the cumulative incidence of industry general payments in any amount and payments of $5000 or more per year in the first 6 years of independent physician practice to recent graduates of Accreditation Council for Graduate Medical Education (ACGME)–accredited residency or fellowship programs in orthopedic surgery and neurosurgery, using internal medicine as a comparison group. We also examined the association of the sex and age of the newly independent physicians in these specialties with the acceptance of general payments.

## Methods

In this cohort study, the selection of physician specialties to study was determined based on the OPP data. For example, active physicians in orthopedic surgery and neurosurgery accepted the highest reported values per physician in the 2015 OPP data.^20^ In addition, physicians in orthopedic surgery and neurosurgery had the highest proportion of physicians (11% and 13%, respectively) accepting more than $10 000 in general payments out of all active physicians who accepted general payments within their specialty.^20^ Meanwhile, internal medicine was chosen as a comparison group because internal medicine accounted for the largest number and percentage of physicians of all specialties in the 2015 OPP data (103 588 of 449 864 [23%]).^20^ Similar trends in industry payments to neurosurgery, orthopedic surgery, and internal medicine physicians persisted in the OPP databases between 2014 and 2018.^1^ The institutional review board at Johns Hopkins University exempted this study from review and waived participant consent because the data for analysis were deidentified. This study followed the Strengthening the Reporting of Observational Studies in Epidemiology (STROBE) reporting guideline for cohort studies.^21^

### ACGME Database of Recent Graduates

The ACGME database of recent graduates included the National Practitioner Identifier number, first name, middle initial, last name, sex, date of birth, graduation year, and specialty or subspecialty of all graduates from ACGME-accredited residency and fellowship programs in orthopedic surgery, neurosurgery, and internal medicine between January 1, 2015, and December 31, 2019. The orthopedic surgery group included recent graduates of ACGME-accredited orthopedic surgery residency programs as well as ACGME-accredited orthopedic surgery fellowship programs in sports medicine, spine surgery, adult reconstructive surgery, hand surgery, trauma surgery, pediatric orthopedic surgery, and foot and ankle surgery. The neurosurgery group included only recent graduates of ACGME-accredited neurosurgery residency programs because there is no ACGME-accredited neurosurgery fellowship program. The internal medicine group included recent graduates of ACGME-accredited internal medicine residency programs as well as ACGME-accredited internal medicine fellowship programs in critical care medicine or pulmonary medicine, cardiac or cardiovascular disease, oncology or hematology, gastroenterology, nephrology, rheumatology, endocrinology, and infectious diseases.

### OPP Database

The combined database of physicians who accepted any general payments between July 1, 2015, and June 30, 2021, in 3 specialties (orthopedic surgery, neurosurgery, and internal medicine) was developed using the CMS general payments data set downloadable files as of August 16, 2022. This data set included all active physicians accepting any general payments from industry.^12^

### Data Linkage

A data use agreement was executed, allowing the ACGME and Johns Hopkins University to securely exchange personal identifying data of the recent graduates. Two data sets (ACGME’s recent graduate data and OPP general payments data) were linked based on a combination of identifying variables, including National Practitioner Identifier number, name, graduation year, and specialty or subspecialty. An aggregate data file for analysis was generated.

Main outcomes were general payments to newly independent physicians, including any general payments and at least $5000 of general payments in aggregate value per year. The first main outcome was selected because physician decision-making can be affected by even small gifts.^22,23^ The second main outcome was selected because the US Department of Health and Human Services currently considers it to be a significant financial conflict of interest if a physician receives aggregated payments exceeding $5000 in 1 year.^24^

### Statistical Analysis

To assess the extent and timing after graduation of the general payments to newly independent physicians, the cumulative incidence of payments was calculated. To assess independent associations between specialty, sex, age, and receipt of general payments, univariable and multivariable Cox proportional hazards regression models were performed, and the hazard ratios (HRs) and 95% CIs for specialties, age, and sex were estimated. The interaction between specialty and sex was assessed by comparing models with and without interaction using the likelihood ratio test.

**Figure 1.**
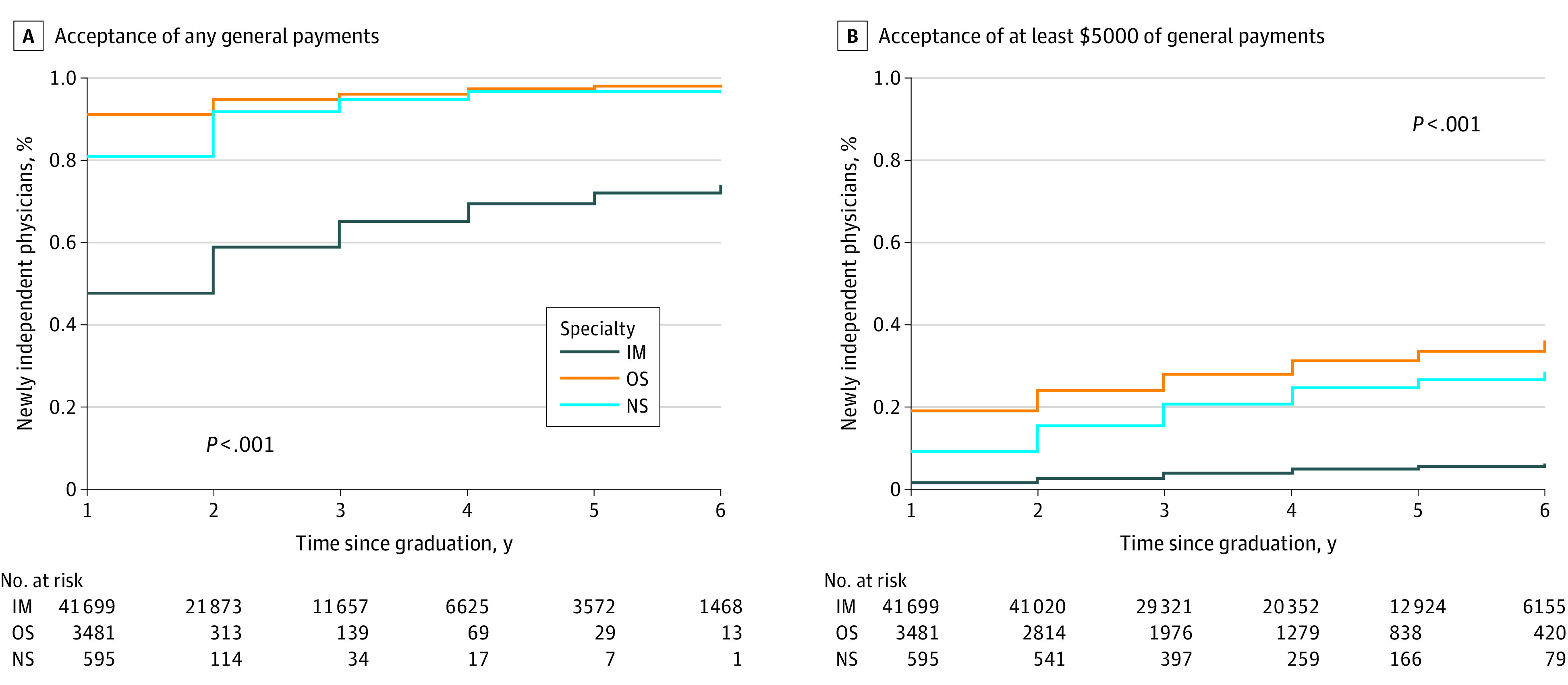
Percentage of Newly Independent Physicians Who Accepted General Payments by Independent Physician Practice Year, Stratified by Specialty IM indicates internal medicine; NS, neurosurgery; and OS, orthopedic surgery.

**Figure 2.**
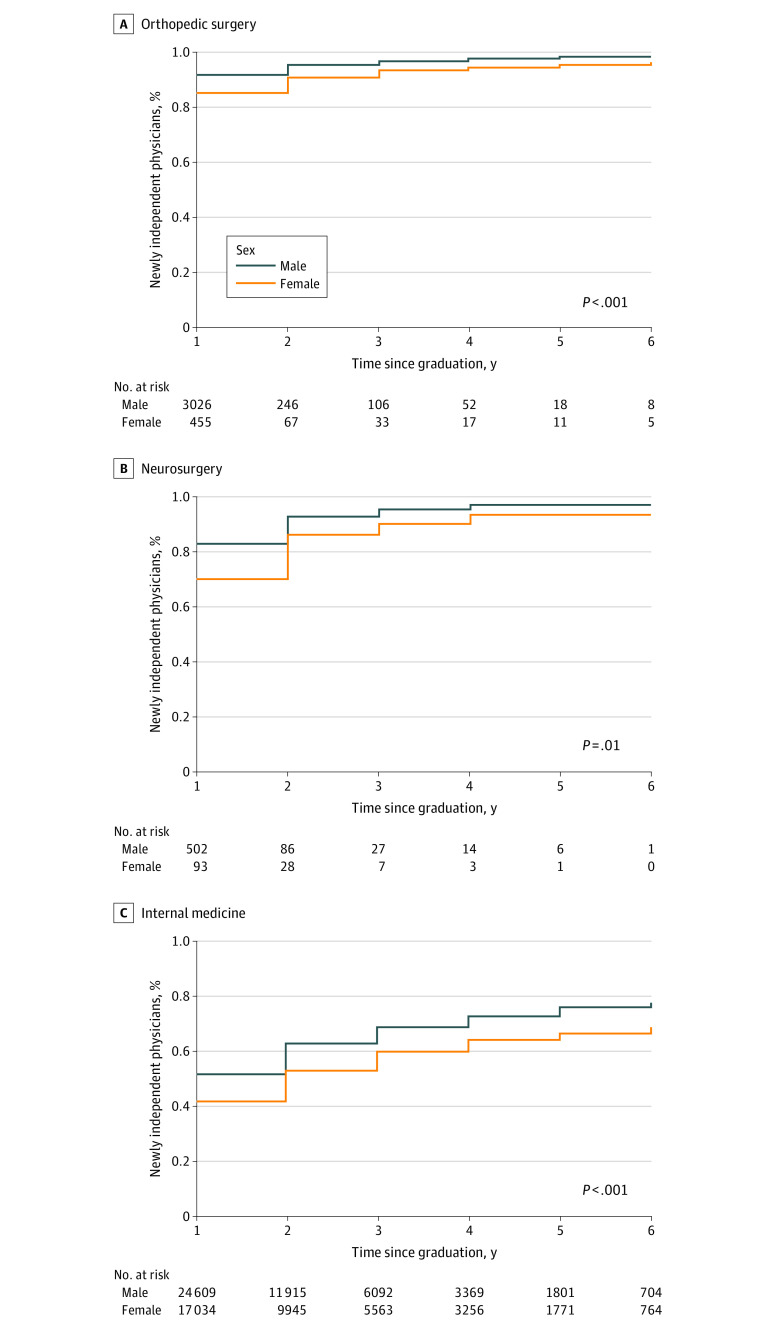
Percentage of Newly Independent Physicians Who Accepted Any General Payments by Independent Physician Practice Year, Stratified by Sex

**Figure 3.**
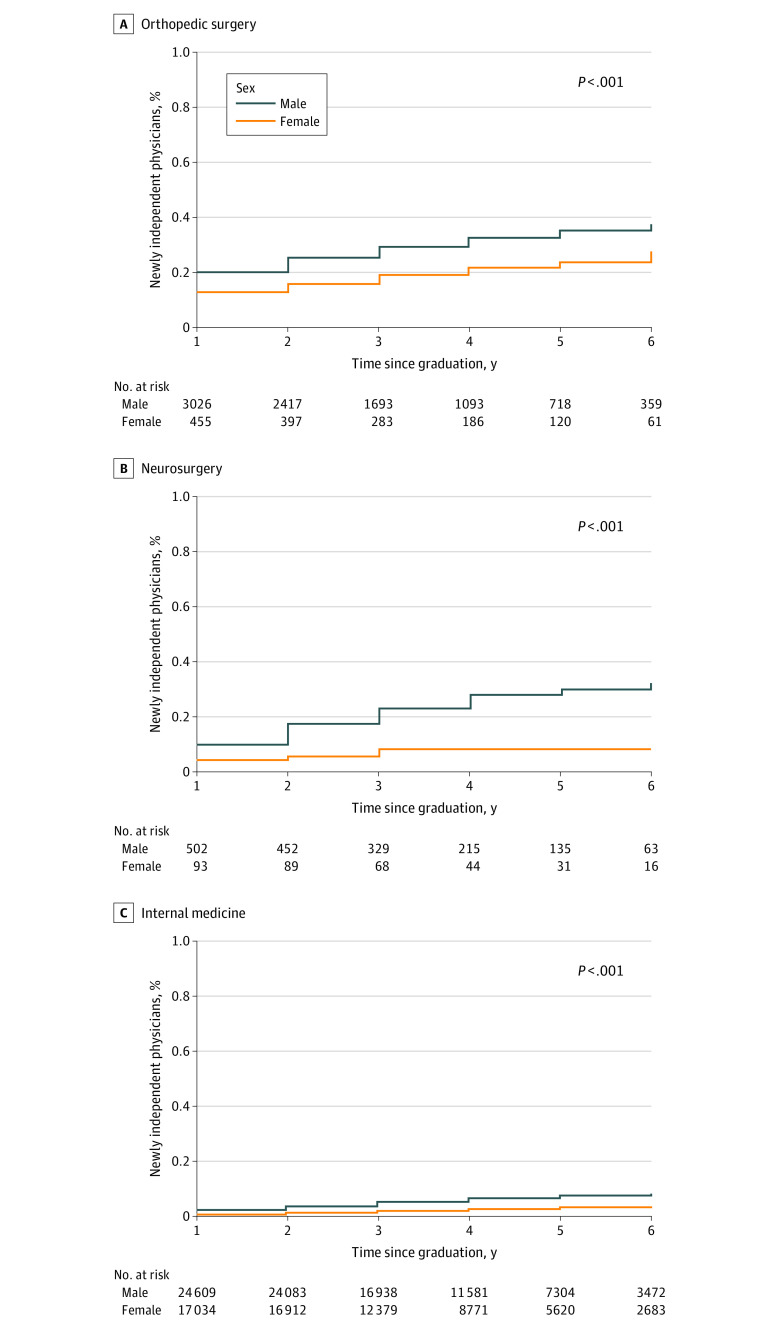
Percentage of Newly Independent Physicians Who Accepted at Least $5000 of General Payments by Independent Physician Practice Year, Stratified by Sex

All statistical analyses were performed using SAS statistical software, version 9.4 (SAS Institute Inc). Two-tailed *P* values were considered statistically significant at *P* < .05. The *P* value for Figure 1, Figure 2, and Figure 3 used the log-rank test.

## Results

There were 45 745 recent graduates (28 137 men [62%]; median age at graduation, 33.0 [IQR, 31.0-35.0 years]) in orthopedic surgery (n = 3481), neurosurgery (n = 595), and internal medicine (n = 41 669) who recently graduated from ACGME-accredited residency or fellowship programs and became newly independent physicians between 2015 and 2019 (Table 1). The age of newly independent physicians was similar between specialties. Data regarding physicians’ race and ethnicity or setting or region of the residency or fellowship program were not available. There were more male physicians (28 137 [62%]) than female physicians (17 582 [38%]) in these 3 specialties, but the disparity was most pronounced for orthopedic surgery (men, 3026 of 3481 [87%]; women, 455 of 3481 [13%]) and neurosurgery (men, 502 of 595 [84%]; women, 93 of 595 [16%]). Overall, 77% of these physicians (n = 35 320) accepted any general payments, while 9% (n = 3998) accepted at least $5000 of general payments per year. More than 88% (31 112 of 35 320) of physicians who accepted any payments accepted more than 1 payment during this period. The overall general payments accepted between July 2015 and June 2021 by these newly independent physicians totaled more than $172 million.

**Table 1.** Demographic Characteristics of the Newly Independent Physicians Who Graduated from ACGME-Accredited Residencies or Fellowships Between 2015 and 2019

Demographic characteristic	Orthopedic surgery (n = 3481)	Neurosurgery (n = 595)	Internal medicine (n = 41 669)^a^	Overall (N = 45 745)
Age at graduation (IQR), y	33.0 (32.0-35.0)	34.0 (33.0-37.0)	33.0 (31.0-35.0)	33.0 (31.0-35.0)
Sex, No. (%)				
Female	455 (13)	93 (16)	17 034 (41)	17 582 (38)
Male	3026 (87)	502 (84)	24 609 (59)	28 137 (62)
Final ACGME-accredited training, No. (%)				
Residency	1405 (40)	595 (100)	21 176 (51)	23 176 (51)
Fellowship	2076 (60)	0	20 493 (49)	22 569 (49)
General payment accepted, No. (%)				
Any amount	3423 (98)	582 (98)	31 315 (75)	35 320 (77)
At least $5000	1576 (45)	146 (25)	2276 (5)	3998 (9)
General payments accepted per physician per year, median (IQR), $	1183.95 (267-3332.67)	586.63 (162.59-2012.27)	187.92 (67.56-642.72)	229.43 (80.05-895.08)
Total general payments accepted between July 2015 and June 2021, $	47 652 767.69	6 754 124.61	117 860 948.79	172 267 841.09

Abbreviation: ACGME, Accreditation Council for Graduate Medical Education.

^a^ Twenty-six physicians in the internal medicine group did not state their sex.

### Cumulative Incidence Analyses

Using the linked data set, we performed a cumulative incidence analysis of recent graduates in orthopedic surgery, neurosurgery, and internal medicine accepting any general payments and accepting at least $5000 of general payments per year after their graduation from residency. Higher percentages of the newly independent physicians in orthopedic surgery and neurosurgery accepted any general payments and at least $5000 of general payments per year compared with those in internal medicine, reflecting a similar pattern observed for all active physicians in those specialties.^20^ In the first 2 years of independent practice, 95% (3297 of 3481), 92% (546 of 595), and 59% (24 522 of 41 699) of newly independent physicians in orthopedic surgery, neurosurgery, and internal medicine, respectively, accepted any general payments (Figure 1A). In terms of significant financial conflicts of interest in the first 2 years of independent practice, 24% (834 of 3481), 15% (92 of 595), and 3% (1093 of 41 699) of newly independent physicians in orthopedic surgery, neurosurgery, and internal medicine, respectively, accepted at least $5000 of general payments per year (Figure 1B).

The percentage of physicians accepting general payments steadily increased during the first 6 years of independent practice, indicating the continual development of financial relationships and significant financial conflicts of interest between newly independent physicians and industry. In the first 2 years of independent practice, a higher percentage of male physicians than female physicians accepted any general payments (orthopedic surgery, 2884 of 3026 [95%] vs 413 of 455 [91%]; *P* < .001; neurosurgery, 466 of 502 [93%] vs 80 of 93 [86%]; *P* = .01; and internal medicine, 15 462 of 24 609 [63%] vs 9043 of 17 034 [53%]; *P* < .001) (Figure 2) and at least $5000 of general payments (orthopedic surgery, 763 of 3026 [25%] vs 71 of 455 [16%]; *P* < .001; neurosurgery, 87 of 502 [17%] vs 5 of 93 [5%]; *P* < .001; and internal medicine, 882 of 24 609 [4%] vs 210 of 17 034 [1%]; *P* < .001) (Figure 3) in all 3 specialties. All sex differences were statistically significant (*P* < .01 for all).

### Cox Proportional Hazards Regression Analyses

Hazard ratios for receipt of general payments were calculated in univariable and multivariable analyses, confirming the findings from the cumulative incidence analyses (Table 2). In multivariable analyses, there was significant interaction between specialty and sex on accepting both any and at least $5000 of general payments. The HRs of accepting any general payments per year among specialties were different between sexes. For women, the HR was 5.36 (95% CI, 4.42-6.51) for orthopedic surgery vs internal medicine and 3.25 (95% CI, 2.24-4.72) for neurosurgery vs internal medicine. For men, the HR was 7.01 (95% CI, 6.35-7.73) for orthopedic surgery vs internal medicine and 4.08 (95% CI, 3.37-4.94) for neurosurgery vs internal medicine (*P* = .03). The HRs for accepting at least $5000 of general payments per year among specialties were also different between sexes. For women, the HR was 10.24 (95% CI, 8.13-12.89) for orthopedic surgery vs internal medicine and 3.14 (95% CI, 1.47-6.68) for neurosurgery vs internal medicine. For men, the HR was 6.76 (95% CI, 6.20-7.36) for orthopedic surgery vs internal medicine and 4.80 (95% CI, 3.98-5.80) for neurosurgery vs internal medicine (*P* = .002).

**Table 2.** Hazard Ratios of Accepting Any or at Least $5000 General Payments From Univariable and Multivariable Analyses

Receiving any general payments^a^						Accepting at least $5000 of general payments^b^					
Univariable			Multivariable			Univariable			Multivariable		
Risk factor	HR (95% CI)	*P* value	Risk factor	HR (95% CI)	*P* value	Risk factor	HR (95% CI)	*P* value	Risk factor	HR (95% CI)	*P* value
Specialty			Female sex			Specialty			Female sex		
Internal medicine	1 [Reference]	<.001	Internal medicine	1 [Reference]	.03	Internal medicine	1 [Reference]	<.001	Internal medicine	1 [Reference]	.002
Orthopedic surgery	7.31 (6.70-7.97)		Orthopedic surgery	5.36 (4.42-6.51)		Orthopedic surgery	8.51 (7.87-9.22)		Orthopedic surgery	10.24 (8.13-12.89)	
Neurosurgery	4.46 (3.77-5.29)		Neurosurgery	3.25 (2.24-4.72)		Neurosurgery	5.83 (4.87-6.99)		Neurosurgery	3.14 (1.47-6.68)	
Sex			Male sex			Sex			Male sex		
Female	1 [Reference]	<.001	Internal medicine	1 [Reference]	.03	Female	1 [Reference]	<.001	Internal medicine	1 [Reference]	.002
Male	1.53 (1.49-1.58)		Orthopedic surgery	7.01 (6.35-7.73)		Male	3.26 (2.96-3.59)		Orthopedic surgery	6.76 (6.20-7.36)	
NA	NA		Neurosurgery	4.08 (3.37-4.94)		NA	NA		Neurosurgery	4.80 (3.98-5.80)	
Age at graduation, per 5 y	1.26 (1.24-1.29)	<.001	Age at graduation, per 5 y	1.23 (1.21-1.25)	<.001	Age at graduation, per 5 y	1.24 (1.19-1.29)	<.001	Age at graduation, per 5 y	1.21 (1.15-1.26)	<.001

Abbreviations: HR, hazard ratio; NA, not applicable.

^a^ Likelihood ratio test for comparing models with or without interaction between sex and specialty group on receiving any general payments: χ^2^_2_ = 6.76 (*P* = .03).

^b^ Likelihood ratio test for comparing models with or without interaction between sex and specialty group on receiving at least $5000 of general payments: χ^2^_2_ = 12.17 (*P* = .002).

Older physicians were more likely to accept any general payments (HR per 5 years, 1.23 [95% CI, 1.21-1.25]; *P* < .001) and at least $5000 of general payments (HR per 5 years, 1.21 [95% CI, 1.15-1.26]; *P* < .001) (Table 2). The multivariable HRs for accepting any general payments for specialties are only slightly smaller than the univariable HRs, indicating that the lower payments to internal medicine physicians are not primarily owing to confounding by sex.

## Discussion

A conflict of interest in medicine exists when a physician’s ability to act in the best interests of a patient could be influenced by relationships with others.^25^ For example, a potential conflict of interest exists when the introduction of a new therapy or device coincides with higher industry payments to physicians.^26,27^ A conflict of interest likely exists when there is an association between the receipt of payments for nonresearch compensation purposes and increased medication prescription.^22,28^ Because of growing concerns that the wide-ranging financial ties between medical professionals and industry may disproportionately affect professional judgments and the goals of medicine, the Institute of Medicine, the predecessor to the National Academy of Medicine, advocated for the medical community to identify, limit, and manage potential conflicts of interest.^4^ In this study, we examined the extent of and timing for the formation of new financial relationships and potential conflicts of interest between industry and physicians who recently graduated from ACGME-accredited residency or fellowship programs in orthopedic surgery, neurosurgery, and internal medicine. There are several important observations in this study regarding potential conflicts of interest in the form of general payments from industry to newly independent physicians. First, the financial relationship between these newly independent physicians and industry began to develop in the first year after graduation from their training programs and continued to expand in the early years of independent physician practice. Second, the recent graduates in surgical specialties (orthopedic surgery and neurosurgery) accepted higher general payments compared with recent graduates in internal medicine. Third, newly independent male physicians accepted higher general payments compared with their female colleagues in all 3 specialties.

Several studies on the OPP demonstrated significant financial relationships and potential conflicts of interest between industry and active physicians in the US.^1,5,6,17,29,30,31,32,33,34,35,36,37^ For example, in 2015, approximately 48% of all active US physicians accepted a total of $2.4 billion in industry-related payments ($1.8 billion in general payments, $75 million for research payments, and $544 million for ownership or investment interests), with a higher likelihood and higher value of payments to physicians in surgical specialties than to those in primary care specialties and to male than to female physicians.^20^ However, most studies on the OPP so far have examined the prevalence of financial relationships between industry and all active physicians, both established and newly independent physicians. By combining the CMS’s OPP data and the ACGME’s recent graduate data to study cumulative incidence, we were able to demonstrate for the first time, to our knowledge, that the financial relationship between industry and newly independent physicians commenced and expanded in the early years of independent physician practice for a significant proportion of new graduates in 3 specialties.

Similar to the pattern seen in the studies on the OPP to all active physicians,^17,19,20^ we found that surgical specialists and male physicians accepted higher general payments, even in their early years of independent practice. It is unknown whether industry offered more payment opportunities to these physicians, these physicians sought more opportunities for industry payment, or both. The common practice of accepting industry payments by more established colleagues may have influenced the similar practice by newly independent physicians.^38^ In addition, it is possible that physicians who have higher educational debt are more inclined to accept nonresearch or noninvestment general payments from industry.^39^ For example, there are some differences in the median educational debt of graduating medical students by their intended specialty (eg, internal medicine, $175 000; orthopedic surgery, $190 000; neurosurgery, $187 500).^40^ However, mean annual physician compensation differs significantly between specialties, ranging from $248 000 for physicians in internal medicine to more than $500 000 for those in neurosurgery or orthopedic surgery.^41^ In addition, significant sex-based income disparities still exist, with male physicians earning higher incomes than female colleagues regardless of their specialty.^41^ Thus, it is unclear why surgical specialists and male physicians accepted higher general payments in their early independent practice.

It will be intriguing to explore the potential factors associated with acceptance of general payments by newly independent physicians, including the year of completing residency training, training program–level factors, and resident evaluation measures, such as the ACGME’s Milestones in related Core Competencies.^42^ As a Core Competency in graduate medical education (GME), professionalism assesses one’s integrity, respect for others, responsiveness to patient needs that supersedes self-interest, and accountability to patients, society, and the profession.^42^ Systems-based practice, another Core Competency in GME, measures one’s ability to work effectively in various health care settings with appropriate considerations of cost-benefit analyses and pharmaceutical and medical device costs.^42^ Deficiency in Core Competencies, which can be measured as Milestones in professionalism and systems-based practice, may be associated with the physician’s acceptance of general payments from industry.^42^ To achieve the goal of the medical community in protecting the integrity of professional judgment and preserving public trust, earlier recognition and intervention during GME to improve professionalism and systems-based practice skills may limit future conflicts of interest with industry and improve health care affordability and cost transparency of prescription drugs and medical devices.

### Limitations

There are several potential limitations to this study. First, this study examined the extent and pace of the initial formation of the financial relationship between industry and newly independent physicians in 3 specialties only. It will be important to explore the formation of financial relationship between industry and recent graduates of the rest of medical and surgical specialties. Second, in this study, no distinction was made between graduates from core specialty residencies and graduates from subspecialty fellowships. It will be interesting to investigate the potential role of subspecialties in accepting general payments, for example, in the procedure-focused internal medicine subspecialties vs the rest of internal medicine subspecialties. Third, small gifts from industry to physicians may seem to be inconsequential. However, research suggests that small gifts, even a single meal valued at less than $20, can be associated with unconscious bias in physician decision-making (eg, in medication prescription).^22,23^ Thus, we considered that a physician accepting the first industry payment of any amount as developing a financial relationship with industry that can potentially influence physician behavior.

## Conclusions

This cohort study found that the financial relationship and potential conflicts of interest between newly independent physicians and industry began to form and continued to expand in the early years of independent physician practice. Surgical specialty and male sex of the newly independent physicians were significantly associated with acceptance of higher general payments in the early years of independent practice. Further studies are needed to evaluate whether modifiable factors, such as the ACGME’s Milestone ratings in professionalism and systems-based practice during GME training, can indicate the future outcome of acceptance of general payments during independent physician practice.
